# Extracellular Polymeric Substances: Still Promising Antivirals

**DOI:** 10.3390/v14061337

**Published:** 2022-06-19

**Authors:** Raquel Bello-Morales, Sabina Andreu, Vicente Ruiz-Carpio, Inés Ripa, José Antonio López-Guerrero

**Affiliations:** 1 Departamento de Biología Molecular, Edificio de Biología, Universidad Autónoma de Madrid, Darwin 2, Cantoblanco, 28049 Madrid, Spain; sandreu@cbm.csic.es (S.A.); vicente.ruiz@uam.es (V.R.-C.); ines.ripa@cbm.csic.es (I.R.); ja.lopez@uam.es (J.A.L.-G.); 2 Centro de Biología Molecular Severo Ochoa, Consejo Superior de Investigaciones Científicas (CSIC), Universidad Autónoma de Madrid (UAM), Cantoblanco, 28049 Madrid, Spain

**Keywords:** extracellular polymeric substance, sulfated polysaccharides, polyanions, antivirals, coronaviruses, herpesviruses, SARS-CoV-2

## Abstract

Sulfated polysaccharides and other polyanions have been promising candidates in antiviral research for decades. These substances gained attention as antivirals when they demonstrated a high inhibitory effect in vitro against human immunodeficiency virus (HIV) and other enveloped viruses. However, that initial interest was followed by wide skepticism when in vivo assays refuted the initial results. In this paper we review the use of sulfated polysaccharides, and other polyanions, in antiviral therapy, focusing on extracellular polymeric substances (EPSs). We maintain that, in spite of those early difficulties, the use of polyanions and, specifically, the use of EPSs, in antiviral therapy should be reconsidered. We base our claim in several points. First, early studies showed that the main disadvantage of sulfated polysaccharides and polyanions is their low bioavailability, but this difficulty can be overcome by the use of adequate administration strategies, such as nebulization of aerosols to gain access to respiratory airways. Second, several sulfated polysaccharides and EPSs have demonstrated to be non-toxic in animals. Finally, these macromolecules are non-specific and therefore they might be used against different variants or even different viruses.

## 1. Introduction

Eukaryotic and prokaryotic microorganisms can produce and secrete extracellular polymeric substances (EPSs) that may mediate many aspects of their biology including physical resistance, resilience or adaptation to the environment. Bacterial EPSs have been widely described, but these biopolymers may be also produced by archaea [1,2,3,4], protists [5,6,7] and fungi [8]. EPSs are the set of organic polymeric compounds released by microorganisms into the environment. They can, therefore, be made up of different substances. However, the acronym EPS has also been widely used to designate a specific category of such substances: the exopolysaccharides. In fact, the term EPS has been used to define the extracellular polysaccharides secreted from microorganisms into their surrounding environment in contrast to capsular polysaccharides, which are associated to cell membrane [9,10], although EPSs contain other major components in addition to these polymeric carbohydrates.

The main components of EPSs are carbohydrates and proteins, although lipids [11,12], nucleic acids [13] and humic substances [14] can also be present. However, its chemical composition is highly heterogeneous and variable [15]. Among biopolymers, polysaccharides are the most abundant and varied group and, indeed, they are the most studied components of EPSs [16,17]. Several types of monosaccharides may be present in EPSs: hexoses and deoxy-hexoses, pentoses, uronic acids, or amino sugars such as glucosamine or galactosamine. In addition, several substituents such as sulfates, phosphates, acetates or amino acids, can be linked to the carbohydrate backbone [9]. The negative charge of EPSs is often imputable to the presence of negatively charged sulfate groups and glucuronic acids and, in fact, sulfated polysaccharides such as heparins, heparan sulfates, dextran sulfates or chondroitin sulfates are abundant polysulfates among EPSs [18]. Besides carboxyls, phosphates and sulfates, the negatively charged groups may also include aspartic and glutamic acids [19]. Positively charged groups may be found in amino sugars [20]. The production and chemical composition of EPSs is influenced by several factors, such as species and strain; nutrients and substrate type—carbon sources and carbon/nitrogen ratio—; environmental conditions—pH, temperature, dissolved oxygen, shear force and salinity—; and physiology [21].

On the other hand, the polymeric structure of EPSs gives them properties that single-molecule drugs lack. Given their high molecular weight and their repetitive structure, polymers can be adjusted to regulate their pharmacodynamics and pharmacokinetics [22]. Another property of polymers is their “polyvalency”, that is, multiple repeated units or attached ligands that can simultaneously bind to numerous receptors on the target. That is an interesting property, since polyvalent interactions are habitually stronger than monovalent binding, given that multiple individual ligand–receptor interactions can act synergistically [22].

Here, we have reviewed the antiviral capacity of EPSs, especially of exopolysaccharides, their main carbohydrate fraction. We have also included some general data about the antiviral activity of non-secreted biopolymers.

### 1.1. Biological Functions of EPSs

Since the biosynthesis of EPSs is an energy demanding process, they must provide selective advantages to the microorganisms that secrete them [16,23]. In fact, EPSs may perform significant adaptive functions, from attachment to surfaces leading to the formation of biofilms [24,25] to protection against toxic compounds such as antibiotics, bile salts, lysozyme or metal ions [26,27,28]. As said before, another important function of EPSs include protection from environmental stress, such as changes in pH, temperature or osmolarity, as well as moisture limitation or harmful contaminants [16].

EPSs and, in general, polyanions can facilitate evasion of the immune response or, on the contrary, inhibit viral infections by immunomodulation. On the other hand, EPSs may enhance the transfer of genetic material between microorganisms, and, in fact, the rates of natural bacterial transformation and conjugation seem to be higher within biofilms [16]. For instance, *Campylobacter jejuni*, a naturally competent bacterium for DNA transformation, may transfer antibiotic resistance genes more frequently when it is engulfed in biofilms [29]. Regarding bacteriophages, EPSs may inhibit phage adsorption by providing a physical barrier between the phage and the membrane receptors [30] or, conversely, facilitate infection [31].

In relation to ecological functions, EPSs released by eukaryotic phytoplankton and bacteria in marine environments represent a major constituent to the total dissolved organic carbon (DOC) pool in the oceans. Moreover, the polyanionic nature of EPSs plays important ecological functions in marine systems, including microbial adhesion and biofilm formation [19]. In addition, the EPSs molecules form a three-dimensional structure from which cells may localize extracellular activities and conduct symbiotic interactions that cannot be undertaken efficiently by free-living cells [19]. EPSs are also involved in horizontal transfer of genetic information, and, in the water-column, they contribute to the formation of marine snow, transparent exopolymer particles, sea-surface microlayer biofilm and marine oil snow [19]. Regarding soil ecosystems, EPSs can improve the aggregation of soil particles, thus assisting plants by maintaining environmental moisture and entrapping nutrients [32,33]. This capacity to aggregate soil particles is important for soil moisture, structure and fertility [16,34]. In addition, EPS may play a significant role in the process of symbiosis between plants and nitrogen-fixing rhizobia [16,35,36].

### 1.2. Applications of EPSs

EPSs have been widely used in the food industry as viscous, stabilizing and emulsifying agents or to improve the rheological properties and texture of bread and fermented milk products [28,37,38]. In addition, EPSs have been also used for health applications, given their role as antioxidants or anti-inflammatories [39,40,41].

Regarding environmental applications, EPSs have been used in several areas, including bioremediation or wastewater treatment systems. For instance, EPS-producing *Bacillus licheniformis* strain KX657843, isolated and identified based on 16S rRNA sequencing and phylogenetic analysis, showed significant capacity for plant growth promotion and Cu(II) and Zn(II) removal. The emulsification index of the EPS, (indicator of biosurfactant production), as well as the capacity of this strain to remove metals, suggested a role for this *Bacillus* in bioremediation [42]. In general, knowledge about EPSs potential in pollution control applications is not abundant, and the application of EPSs in water, wastewater and sludge flocculation, dewatering and treatment is still under investigation, so further research is still required before their potential application in field processes [15]. However, preliminary studies have suggested that bacterial polymers might be used for interesting environmental applications in wastewater treatment systems—including the flocculation of secondary wastewater, or as an adsorbent for heavy metal removal from effluents—, soil remediation and soil erosion control [15,43,44,45].

## 2. Antiviral Activity of Polysaccharides and EPSs

Sulfated polysaccharides and EPSs can exert antimicrobial activity [21,26,46], and many studies have reported antiviral effects against viruses, such as herpes simplex type 1 (HSV-1) and 2 (HSV-2) [47,48,49], pseudorabies virus (PRV) and vesicular stomatitis virus (VSV) [49], encephalomyocarditis virus (EMCV) [50], influenza virus [51], infectious hematopoietic necrosis virus (IHNV), rotaviruses [52], African swine fever virus (ASFV) [53] and infectious pancreatic necrosis virus (IPNV) [54]. In fact, EPSs have been proposed as new promising therapeutic drugs [17].

The antiviral effects of polysaccharides were reported many decades ago [18]. In 1947, the first report describing the antiviral activity of polysaccharides was published [55] and several years later, the ability of heparin and other polysaccharides as HSV-1 inhibitors was also demonstrated [56,57,58]. Currently, several sulfated polysaccharides from algae, cyanobacteria and animals have been described, showing potent inhibitory effects against several human and animal viruses [59]. Early studies reported antiviral effect of algal polysaccharides against mumps and influenza B viruses [60,61]. Later, other marine polysaccharides extracted from Rhodophyta algae were found to be antiviral against HSV-1 and HSV-2 and coxsackievirus B5 [62]. A sulfated polysaccharide isolated from *Arthrospira platensis* inhibited several viruses, including HSV-1, human citomegalovirus (HCMV), influenza A, coxsackievirus, the human immunodeficiency virus (HIV), measles, polio and mumps viruses [61]. Later reports showed that extracts of ten other red algae exerted antiviral effects against HSV-1 and HSV-2, vaccinia virus and VSV [63], although the antiviral activity was prophylactic but not therapeutic. Sulfated polysaccharides from the red alga *Schizymenia pacifica* were also reported to be antiviral against HIV reverse transcriptase and viral replication in vitro [64]. In addition, several polysaccharides from algae, bacteria or fungi, including EPSs produced by lactic acid bacteria (LAB), have been considered as GRAS (generally recognized as safe) by the US FDA, opening interesting possibilities for therapeutics or food supplements [65].

Other important characteristics and structural motifs influencing the antiviral activity of EPSs include molecular weight; aldehyde, carboxyl and methyl groups; uronic acid content; phosphates and sulfate group per sugar residue; branched-chain length; and polyanionic nature [66]. In general, enveloped viruses are more sensitive to polyanionic antivirals than non-enveloped viruses. On the other hand, in general, the higher the molecular weight, the higher the antiviral activity [59,65,67]. However, although the molecular weight of polysaccharides and EPSs often correlates with their antiviral effect, molecular weight can play a dual role, or even have no influence at all. For instance, the antiviral capacity of several semisynthetic and natural sulfated polysaccharides, including agarans, carrageenans and fucans, has shown to correlate with their molecular weight [59]. However, some low-molecular-weight polysaccharides can also generate strong antiviral activity, especially when their sulfate content is high; in addition, low-molecular-weight compounds can inhibit cell-to-cell viral spread more efficiently [59]. Low-molecular-weight compounds can inhibit cell-to-cell spread of viruses more efficiently because polysaccharides with low molecular weight can pass more easily through target cells to act inside them [59,65]. In addition, low-molecular-weight EPSs can stimulate the immune system more effectively [41]. In some cases, the antiviral activity is not consistently related to its molecular weight [68,69].

Since negatively charged sulfated groups can be involved in antiviral efficiency, the degree of sulfation present in EPSs is implicated in their antiviral capacity and, in addition, the specific position of the sulfate ester group is also important for the antiviral activity of sulfated polysaccharides [59,65,70,71,72,73]. On the other hand, disparities in the viral envelope glycoproteins may explain the different susceptibility of enveloped viruses to EPSs [18].

### 2.1. Mechanisms of Action

**Figure 1:**
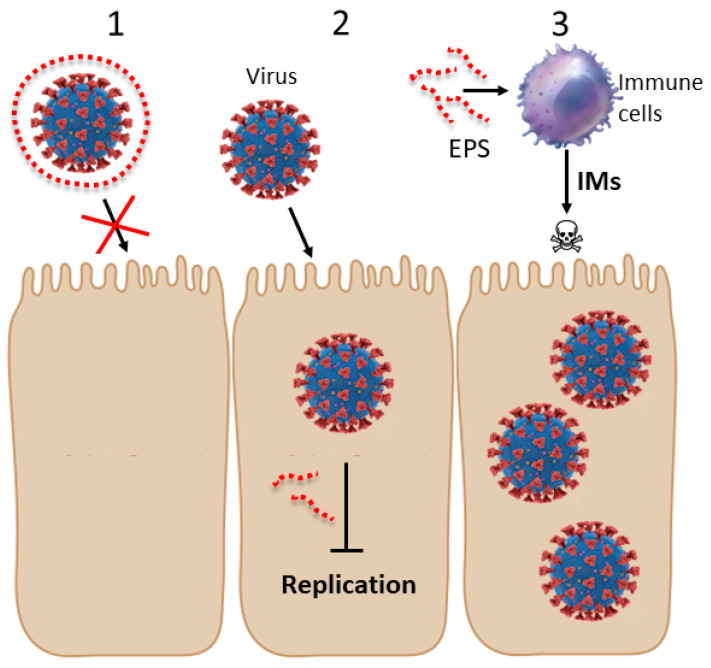
**Mechanisms of antiviral action of microbial exopolymers.** EPSs can exert their antiviral activity via different mechanisms. Virucidal agents act by inactivating viruses. Inactivation can occur not only by damaging the virions, but also by blocking them and impeding the adsorption of virions to cells (red cross). Some EPSs can wrap the virions via electrostatic interactions, thus preventing viral adsorption and, therefore, exerting a virucidal effect (**1**). Other EPSs exert an antiviral effect allowing the viral entry but later impeding the viral replication. Therefore, antivirals inhibit replication of viable viruses in the cells (**2**). EPSs can also exert antiviral effect by activating immune cells and inducing them to secrete immunomodulators (IMs) and to kill virus-infected cells (**3**).

The antiviral mechanisms of exopolymers can operate in many different ways: they can inactivate viruses; inhibit viral adsorption, entry or replication; or trigger the activation of the immune system [74] (Figure 1).

To date, most EPSs affect viral infections via a virucidal effect by impeding attachment. Viral entry is the process that follows viral attachment and triggers the delivery of the viral genome into the cell. Viral receptors are host cell molecules on the plasma membrane, mostly glycoproteins, that bind virions and are essential for viral entry and subsequent virus infection. Viral receptors play physiological roles for the cell functions that have nothing to do with infection, and viruses commonly bind to their cell receptors with higher affinity than they do to attachment factors, which merely support virus binding. On the contrary, viral receptors either induce conformational changes in viral proteins that are required for viral entry and/or result in delivery of virions to cellular domains or compartments required for entry [75]. This difference explains why receptors are required for viral infection whereas attachment factors are not. Thus, if the deletion of a host molecule from an animal model or a cell type prevents infection, then it can be considered a viral receptor (although, it should be noted that some viruses may use more than one viral receptor).

#### 2.1.1. Virucidal Effect

Antiviral agents are chemical compounds, usually drugs, which inhibit the proliferation of viruses. Virucidal agents, in contrast, are physical or chemical agents, such as disinfectants, that specifically can irreversibly inactivate or destroy viruses, thus impeding them to enter host cells [76]. Therefore, virucidal agents act when they are added to viruses before viral attachment, hindering subsequent viral adsorption and entry. Several EPSs have a virucidal effect, thus they act by inactivating viruses. For instance, previous experiments carried out in our laboratory demonstrated the capacity of an EPS from *Bacillus licheniformis* IDN-EC to inhibit several enveloped viruses infection in a dose-dependent manner [49]. We demonstrated a dose-dependent virucidal effect when EPS was added to viruses before viral attachment. Our group found out that EPS produced by this strain of *B. licheniformis* exerted a non-specific virucidal effect against HSV-1 and HSV-2, pseudorabies virus (PRV) and VSV, whereas the EPS had no affect against non-enveloped minute virus of mice (MVM). We also demonstrated that the EPS blocked infection before viral entry. This EPS was also shown to be non-toxic in mice.

**Figure 2:**
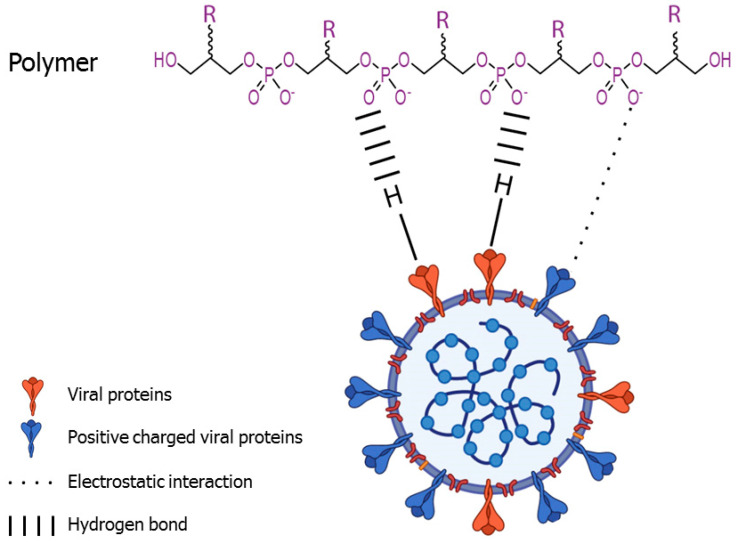
**Schematic diagram representing the mechanistic basis of the inhibitory effect of EPSs and other antiviral polyanions on viral adsorption**. The figure represents a prototypical polymer interacting with a SARS-CoV-2 virion. The main inhibitory mechanisms include non-covalent and non-specific electrostatic interaction between the negatively charged moieties of the EPSs and the positively charged viral glycoproteins. Viral glycoproteins might also interact with H-bond acceptors of the polymer.

Early reports demonstrated that binding of negatively charged polymers with positively charged groups on the HSV-1 viral membrane inhibited the viral attachment to the negatively charged cell membrane [77]. In line with these and other reports, our data suggested that the inhibitory effect of the EPS from *Bacillus licheniformis* might be due to non-covalent and non-specific electrostatic interactions between negatively charged moieties of the EPS and positively charged glycoproteins on the virion surface, blocking the viral glycoproteins and thus preventing binding and viral entry (Figure 2). Various evidence supports this hypothesis: first of all, the nature of the interaction between the EPS and viruses seems to be non-specific, since the polymer is virucidal against several viruses from different families that use different cell receptors [49]. In addition, previous studies have established that polyanionic substances such as sulfated polysaccharides may inhibit enveloped viruses, and that this inhibitory effect may be due to non-covalent interactions [78]. For example, the antiviral effect of the sulfated polysaccharides chondroitin sulfate and dextran sulfate—which inhibited HSV-1 and HIV even when present only during adsorption [79]—has been attributed to an interaction between negatively charged polymers and positive charges on the virions. In fact, the partial inhibition of severe acute respiratory syndrome coronavirus (SARS-CoV) by heparin suggested that the viral envelope might include positively charged clusters which could interact with the negatively charged moieties of the polymer [80]. However, electrostatic forces are not the only factor mediating virus-cell interactions, and, thus, van der Waals forces, hydrophobic effects, cation bridging or steric interactions, may also play a relevant role in virus interactions with the surrounding environment [81]. Viral glycoproteins might also interact with H-bond acceptors on the polymer [78] (Figure 2). In addition, the effect derived from interactions of the polymer with membrane lipids cannot be ruled out.

Carrageenans, one of the major components of red seaweed cell walls and currently the most widely investigated red algae-sulfated polysaccharides [82], are also virucidal against HSV-1 and other enveloped viruses. Carrageenans might inhibit viral infection by direct interaction with the viral envelope by means of its negative charge [82]. In fact, the anti-herpetic effect of carrageenans has been widely demonstrated [83,84,85]. Their direct virucidal effect may be due to the formation of irreversible stable complexes with virions, which block the sites on the viral envelope required for virus attachment to cells [86].

Another virucidal polysaccharide, chitosan (a partially deacetylated polymer of N-acetylglucosamine), can reduce the infectivity of two human enteric viral surrogates: feline calicivirus F-9 (FCV-F9) and bacteriophage MS2 (MS2) [87,88]. The antimicrobial properties of chitosan, a biopolymer obtained by N-deacetylation of chitin, have been known for years [89]. This linear polysaccharide composed of randomly distributed β-(1 → 4)-linked D-glucosamine and N-acetyl-D-glucosamine is antimicrobial against fungi (for instance, *Rhizoctonia solani* and *Candida* spp.) and several Gram-positive and Gram-negative bacteria, including *Staphylococcus aureus*, *Streptococcus* spp., *Enterobacter faecalis*, *Bacillus subtilis*, *Salmonella* Typhimurium or *Escherichia coli* [90]. The mechanism of action can be explained by a process of cell membrane disruption, given that chitosan is a cationic polymer that may interact with the negatively charged cell surface [91]. However, other mechanisms of action have been proposed, including metal chelation, enzyme denaturation and interaction with phosphate groups of nucleic acids [89,91].

Besides this antimicrobial activity, chitosan has been shown to exert antiviral activity against bacteriophages and plant viruses [92]. Regarding animal viruses, chitosan has been used for its properties as an immunomodulator, and its antiviral activity against several human viruses, including HSV-1, H7N9 and H1N1 influenza A viruses; HIV; SARS-CoV-2; and HCMV and Rift Valley fever virus (RVFV) [90,92]. Chitosan has also been used in the development of antiviral vaccines due to its adjuvant properties [93] and in the development of nanoparticle vaccines to treat arboviral infections [94]. Recently, the use of chitosan as a pharmaceutical excipient has been suggested for SARS-CoV-2 treatment [95].

#### 2.1.2. Adsorption

As we have previously stated, virucidal agents act by inactivating viruses. However, inactivation can occur not only by damaging the virions, but also by blocking them and impeding the binding of virions to cells. Therefore, one of the mechanisms by which virucides inactivate viruses is by inhibiting the viral adsorption. Some EPSs can wrap the virions via electrostatic interactions, thus preventing binding and, therefore, exerting a virucidal effect.

Unlike viral receptors, which are essential for infection in vitro and in vivo, viral attachment factors make viral adsorption and infection more efficient, but they are not required for infection [75]. Viral attachment receptors are cell surface molecules that assist virus binding. Interactions between viruses and these attachment factors have low affinity and are relatively nonspecific [75]. Attachment receptors can also act as entry receptors, but are often not sufficient to allow viral infection, and entry receptors are also necessary [96]. However, most of the research on viral adsorption has been done in vitro and data about adsorption in vivo are limited. 

To limit random three-dimensional diffusion and promote the attachment process, many viruses have evolved to attach to the cell glycocalyx, a ubiquitous carbohydrate covering containing negatively charged moieties in which the charge is predominantly contributed by proteoglycans with sulfated glycan side chains termed glycosaminoglycans (GAGs) [96]. There are notable variations in the number of glycan chains, degrees of sulfation and composition of the repeating dissacharide units among the different proteoglycans, resulting in a wide range of charge densities per molecule and giving rise to many different sulfated proteoglycans [96]. 

Several viruses (including members of the *Herpesviridae*, *Adenoviridae*, *Caliciviridae*, *Retroviridae*, *Picornaviridae* and *Flaviviridae* families) as well as other microorganisms, use proteoglycans, mainly heparan sulfates (HSPGs) and chondroitin sulfates (CSPGs), as viral attachment factors. For instance, HSV-1 and HSV-2 attach to cells by binding of the viral proteins gB and gC to the disaccharide repeats of heparan sulfate; foot-and-mouth disease virus (FMDV) also uses HSPGs for binding; regarding HIV, HSPGs bind the positively charged sites in the V3 loop of its surface envelope glycoprotein gp120 [18,96].

Moreover, external pH plays a relevant role on viral adsorption. The electrostatic attraction increases when the viral envelope is oppositely charged to the adsorbent cell surface, but viral proteins in an isoelectric state would not exert strong repulsion or attraction towards cell surface. However, changes in pH can alter such behaviour and changing the pH in relation to the virus isoelectric point may alter the process of adsorption [97].

Regarding therapeutics, few drugs targeting viral adsorption are currently available [96,98,99]. In this context, research on EPSs can open a wide and promising range of possibilities.

#### 2.1.3. Immunomodulation

Polyanions and EPSs can facilitate evasion of the immune response in animals by degrading immunoglobulins and components of the complement system [100,101] but, on the contrary, bacterial polysaccharides may also inhibit viral infections by modulation of the immune response [65,101]. For instance, EPSs produced by *Lactobacillus delbrueckii* [102] or *Streptococcus thermophilus* [103] can activate the Toll-like receptor 3 (TLR3) and the expression of interferon (IFN), which activates other immune cells and may trigger antiviral responses. Furthermore, EPSs can activate NK cells that kill virus-infected cells non-specifically via perforins and granzymes [65].

### 2.2. EPSs with Virucidal or Antiviral Activity

Several microbial species, summarized in Table 1, have been demonstrated to secrete EPSs with antiviral activity. An EPS produced by *Bacillus licheniformis* strain B3-15 (EPS-B3-15) was demonstrated to inhibit HSV-2 infection in peripheral blood mononuclear cells (PBMC) [48]. The antiviral activity seemed to be related to the stimulation of Th1 cytokines (IFN-γ, IFN-α, TNF-α IL-12 and IL-18). A later study [104] analyzed the role of EPS-B3-15 on Th2 cytokine production by PBMC infected or not with HSV-2, finding that EPS-B3-15 treatment was able to control the production of Th2 cytokines, triggering a shift in the balance of cytokine profiles from Th2-type to a Th1-type. An EPS from another strain of *Bacillus licheniformis* [105] was also shown to exert antiviral and immunomodulatory effects against HSV-2. The antiviral effect seemed to be linked to the immunomodulatory activity of the biopolymer, since human PBMC treated with EPS1 produced high levels of Th1 cytokines [106,107]. An EPS from *Geobacillus thermodenitrificans* has also showed immunomodulatory and antiviral effects against HSV-2 [108].

Yogurt fermented with *Lactobacillus delbrueckii* ssp. *bulgaricus* OLL1073R-1 (1073R-1) and the EPS produced by this strain exerted antiviral effects against influenza virus A/PR8 in mice via immunostimulatory effects such as the increase of NK cell activity [109]. The yogurt and EPS were orally administered to BALB/c mice prior to intranasal infection with influenza virus A/PR/8/34 (H1N1), resulting in prolonged survival periods in both the yogurt- and EPS-treated groups compared to controls.

EPSs from the sponge species *Celtodoryx girardae* (global EPS) and from its symbiotic Gram-negative bacteria (bacterial EPS) presented antiviral activity against HSV-1 [110]. The protective effect of EPSs was ascribed to the inhibition of viral adsorption. The Gram-negative genus *Pseudoalteromonas* is another EPS-producing marine microorganism [10]. An EPS obtained from *Pseudoalteromonas* sp., isolated from a sponge, was also demonstrated to exert antiviral effect against HSV-1 [111].

Two different marine strains of the red alga *Porphyridium cruentum* were grown in two different concentrations of sulfate, and the influence of these conditions on the characteristics of the EPS produced was examined, observing that enrichment of the culture medium with sulfate improved the protein and sulfate content of EPS, which displayed a relevant activity against VSV [112].

In a recent study, high molecular weight dextrans synthesized by the LAB *Lactobacillus sakei* MN1 and *Leuconostoc mesenteroides* RTF10 were purified and assayed in infected BF-2 and EPC fish cell-lines for antiviral activity [54]. Both dextrans had significant antiviral activity against the salmonid viruses infectious pancreatic necrosis virus (IPNV) and infectious hematopoietic necrosis virus (IHNV). In vivo assays injecting intraperitoneally the MN1 polymer in rainbow trouts (*Onchorhynchus mykiss*) confirmed the in vitro results, and the increase of IFN-1 and IFN-γ expression revealed immunomodulatory activity [54]. An EPS isolated from *Lactobacillus plantarum* has also been shown to offer protection against rotavirus-induced diarrhea [52].

**Table 1:** EPS-producing microbial species.

EPS/Species/Strain	Inhibited Viruses ^a^	Mechanism ^b^	Ref.
EPS p-KG03 from *Gyrodinium impudicum* KG03	EMCV	AA	[50]
EPS-B3-15 from *Bacillus licheniformis*	HSV-2	IM	[48]
EPS from *Aphanothece halophytica*	Influenza A virus	IM and IVE	[51]
EPS from *Serratia* sp. Gsm01	CMV-Y	AA	[113]
EPS from *Celtodoryx girardae*	HSV-1	IVE	[110]
EPS from *Geobacillus thermodenitrificans*	HSV-2	IM	[108]
EPS TK V3 from *Arthrospira platensis*	Vaccinia virus and ECTV	IVE	[114]
EPS from *Porphyridium purpureum*	Vaccinia virus and ECTV	IVE	[114]
EPS from *Pseudoalteromonas* sp.	HSV-1	IVE	[111]
EPS OLL1073R-1 from *Lactobacillus delbrueckii* ssp. *bulgaricus*	Influenza A virus	IM	[109]
EPS p-KG03 from *Gyrodinium impudicum* KG03	Influenza A virus	AA	[115]
EPS from *Bacillus licheniformis*	HSV-2	IM	[106]
EPS from *Porphyridium cruentum*	VSV		[112]
EPS from *Lactobacillus sakei* MN1	IPNV and IHNV	IM	[54]
EPS from *Leuconostoc mesenteroides* RTF10	IPNV and IHNV	IM	[54]
EPS from *Bacillus horneckiae* APA	HSV-2	IM and AA	[116]
EPS from *Bacillus horneckiae* APA	HSV-1	IM and AA	[47]
EPS from *Lactobacillus plantarum* LRCC5310	Human rotavirus strain WA	IM and IVE	[52]
EPSp from *Bacillus licheniformis* IDN-EC	HSV-1, HSV-2, PRV, VSV	IVE	[49]

^a^ Inhibited viruses: EMCV, encephalomyocarditis virus; HSV-2, herpes simplex virus type 2; CMV-Y, yellow strain of cucumber mosaic virus; HSV-1, herpes simplex virus type 1; ECTV, ectromelia virus; VSV, vesicular stomatitis virus; IPNV, infectious pancreatic necrosis virus; IHNV, infectious hematopoietic necrosis virus: PRV, pseudorabies virus. ^b^ Mechanism of action: AA, antiviral activity; IM, immunomodulation; IVE, inhibition of viral entry.

A halophilous cyanobacterium, *Aphanothece halophytica*, was another microorganism capable of producing an EPS with antiviral activity. That EPS, a sulfated exopolysaccharide, inhibited pneumonia in influenza virus A FM (H1N1)-infected mice. The mechanism of action seemed to be mediated by two main processes. First, by the modulation of the host immune system: enhancement on lymphocyte proliferation, release of IL-1 and IL-2, potentiation of the phagocytic activity of the reticuloendothelial system and improvement of the cytolytic activity of NK cells; and, second, via the interaction of negative charges in EPS with positive charges in the viral envelope [51]. The anti-vaccinia virus activities of the exopolysaccharides isolated from the cyanobacterium *Arthrospira platensis* and the Rhodophyta alga *Porphyridium purpureum* have also been demonstrated [114].

An exopolymer produced by the marine thermotolerant *Bacillus horneckiae* strain APA, of shallow marine vent origin, was also shown to possess antiviral activity. This EPS exerted antiviral and immunomodulatory activities against HSV-2 [116]. A posterior report showed that the EPS also inhibited HSV-1 infection in the very early phase of viral replication, produced low cytotoxicity and activated innate immune response, stimulating both TNF-α and IL-1β gene transcription via NF-kB activation [47].

A sulfated exopolysaccharide, named p-KG03, secreted by the dinoflagellate *Gyrodinium impudicum* strain KG03, also exhibited notable antiviral activity in vitro against EMCV [50]. In a subsequent report, this sulfated EPS demonstrated antiviral activity against influenza A virus, by interfering with viral adsorption and internalization steps [115]. 

Regarding plant viruses, an EPS from a *Serratia* sp. strain Gsm01 showed antiviral activity against a yellow strain of cucumber mosaic virus (CMV-Y). Thus, the spray treatment of tobacco plants using that EPS induced systemic protection against CMV-Y [113].

## 3. The Decline of Sulfated Polysaccharides in Antiviral Research

### 3.1. HIV

It has been known for a long time that heparin and other sulfated polysaccharides are potent and selective inhibitors of HIV replication in cell cultures [18]. Other enveloped viruses, including HSV-1, HSV-2 and HCMV, can also be inhibited by these polysaccharides. When this was initially demonstrated, sulfated polysaccharides appeared as promising candidates in antiviral research. Early inhibitors such as suramin, a polysulfonated compound, were shown to block viral adsorption nonspecifically [117,118]. Later polyanionic compounds effective against HIV were dextran sulfate and heparin [117,119]. Polyanionic HIV inhibitors are generally considered entry inhibitors [117]. These polysaccharides interfered with the fusion process, being the inhibition of virus–cell fusion, the cause of the antiviral activity. On the other hand, antiviral activity of sulfated polysaccharides increased with increasing molecular weight and degree of sulfation [18]. In addition, sulfated polysaccharides displayed interesting properties: they were able to block HIV replication in cell cultures at low concentrations without cytotoxicity; prevented HIV-induced syncytium formation between HIV-infected and normal CD4 T lymphocytes, a mechanism that can drastically enhance HIV infectivity; showed a broad-spectrum antiviral activity against enveloped viruses; and induced low viral drug-resistance [18].

However, the use of polyanionic compounds as systemic agents to combat HIV infection seems to be currently an unfeasible option, although this type of antiviral is still being considered for topical uses [117]. What led to this abandon? An undesirable side-effect of anionic polymers is their recognized anticoagulant activity that would limit therapeutically administrable doses in clinics [120]. However, the major problem of sulfated polysaccharides as antivirals is related to their poor bioavailability. Although the in vitro inhibition of viral infections was confirmed when polyanions were added to the cell cultures, inhibition of enveloped viruses by polyanions was not corroborated in vivo, a failure probably due to complexation of polyanion molecules by cationic blood elements [121]. This fact led virologists to abandon the research of polyanions as antivirals against enveloped viruses, including coronaviruses and currently, in particular, SARS-CoV-2.

### 3.2. SARS-CoV-2

The early success of polyanions in antiviral research in vitro was followed by a later abandonment when in vivo assays disproved the initial in vitro results. However, under our point of view, this rejection should be reconsidered, since this human coronavirus might be attacked directly in the airways, given that initial infection starts mostly there. For that reason, it has been claimed that polysulfates might be used against SARS-CoV-2 before they reach the lungs and other target cells present in the respiratory airways [121]. 

**Figure 3:**
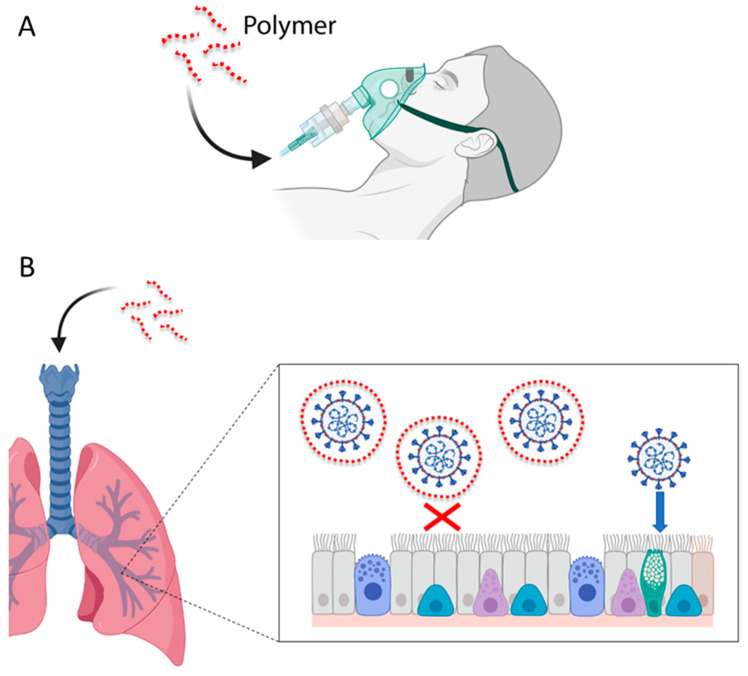
**Alternative routes of administration of EPSs and other polyanions**. (**A**) To overcome the low bioavailability of EPSs, new routes of administration should be tested, especially nebulization of aerosols to access pulmonary alveoli. (**B**) Aerial virions can be trapped by EPSs before attachment in the respiratory epithelium. To enter cells, viral glycoproteins must first attach to the host cell receptors. EPSs may block the viral glycoproteins and prevent viral entry (red cross).

Inhibition of viruses by polyanions in the respiratory tract is not based on the immune system machinery located in blood and mucosas, but on chemical and physicochemical processes. The antiviral inhibitory interaction is electrostatic, non-specific and fast and leads to polyelectrolyte complexes between oppositely charged macromolecules that are difficult to destabilize [121,122]. The first step in viral infection is the interaction of attachment factors, such as sialic acids or HSPGs polyanions, with viral envelope proteins by long distance electrostatic interactions, before interacting with viral entry receptors via short distance contacts. This moment, prior to attachment, is ideal for polysulfates to trap aerial coronaviruses at the level of airways on the basis of the physical-chemistry of polyelectrolyte complexes [121]. The main disadvantage of sulfated polysaccharides and other polyanions, their low bioavailability, can be circumvented by using adequate administration strategies (Figure 3), such as gargling and spraying of an aqueous solution to access oral and nasal cavities, or nebulization of aerosols to access pulmonary alveoli [121]. In addition to these new administration routes, new drug delivery systems by alternative approaches should be assayed. To become therapeutically useful, antiviral agents could be also tested in combination with drug delivery systems such as nanoparticles, liposomes, lipophilic drug derivatives or polymeric lipo-polyethylenimines.

Among the potential concerns regarding the use of polyanions—including sulfated polysaccharides and EPSs—to trap aerial viruses, two can be highlighted [121]. The first one might be the lack of biocompatibility. However, most of the tested polysulfates including heparins, heparan, dextran or chondroitin sulfates, fucoidans, etc. have demonstrated to be very well tolerated after intramuscular, intravenous [121] or intraperitoneal [49] administration. The second one involves the possible interaction of the biopolymers with components of nasal, oral and bronchial secretions, especially with mucins, which must stay neutral and under a gel state [121,123,124]. In this respect, although the risk of inhibition by polysulfates is expected to be low, compatibility and retention of activity must be tested using aerial secretions [121]. 

The use of polyanions including sulfated polysaccharides as therapeutical tools have been investigated and proposed for SARS-CoV-2 treatment [66,90,125,126]. A recent study demonstrated that heparin has an excellent binding affinity to the spike protein (S-protein) of SARS-CoV-2 and, in fact, the S-protein of SARS-CoV-2 attached more strongly to immobilized heparin than the S-proteins of either SARS-CoV or Middle East respiratory syndrome coronavirus (MERS-CoV) [127]. After that, the in vitro antiviral properties of heparin and other related polysaccharides by infecting Vero cells with SARS-CoV-2 in the presence of variable doses of polysaccharides were demonstrated. Results revealed that specific sulfated polysaccharides can bind tightly to the S-protein, suggesting that they can interfere with S-protein binding to the heparan sulfate in host tissues, thus inhibiting viral infection [128]. 

## 4. Conclusions

Sulfated polysaccharides and other polyanions, including EPSs, have been promising candidates in antiviral research for decades. They have demonstrated to be strong antivirals in vitro, they are non-toxic in animals and they are non-specific, which opens the possibility to fight against different pathogens. However, these macromolecules have a major disadvantage: their low bioavailability. This fact led virologists to abandon the research of polyanions as antivirals, but this difficulty can be overcome by using adequate administration strategies, such as nebulization of aerosols to access respiratory airways. Antiviral research on EPSs should consider new routes of administration by alternative approaches, as well as their use in combination with drug delivery systems (such as nanoparticles, liposomes, lipophilic drug derivatives or polymeric lipo-polyethylenimines) to become therapeutically useful antiviral agents.

## Data Availability

Not applicable.
